# Structural and Computational Studies of the SARS-CoV-2 Spike Protein Binding Mechanisms with Nanobodies: From Structure and Dynamics to Avidity-Driven Nanobody Engineering

**DOI:** 10.3390/ijms23062928

**Published:** 2022-03-08

**Authors:** Gennady Verkhivker

**Affiliations:** 1 Graduate Program in Computational and Data Sciences, Keck Center for Science and Engineering, Schmid College of Science and Technology, Chapman University, Orange, CA 92866, USA; verkhivk@chapman.edu; Tel.: +1-714-516-4586; 2 Department of Biomedical and Pharmaceutical Sciences, Chapman University School of Pharmacy, Irvine, CA 92618, USA

**Keywords:** ACE2 host receptor, molecular dynamics, biophysical methods, mutational scanning, binding energy hotspots, allosteric interactions, signal transmission

## Abstract

Nanobodies provide important advantages over traditional antibodies, including their smaller size and robust biochemical properties such as high thermal stability, high solubility, and the ability to be bioengineered into novel multivalent, multi-specific, and high-affinity molecules, making them a class of emerging powerful therapies against SARS-CoV-2. Recent research efforts on the design, protein engineering, and structure-functional characterization of nanobodies and their binding with SARS-CoV-2 S proteins reflected a growing realization that nanobody combinations can exploit distinct binding epitopes and leverage the intrinsic plasticity of the conformational landscape for the SARS-CoV-2 S protein to produce efficient neutralizing and mutation resistant characteristics. Structural and computational studies have also been instrumental in quantifying the structure, dynamics, and energetics of the SARS-CoV-2 spike protein binding with nanobodies. In this review, a comprehensive analysis of the current structural, biophysical, and computational biology investigations of SARS-CoV-2 S proteins and their complexes with distinct classes of nanobodies targeting different binding sites is presented. The analysis of computational studies is supplemented by an in-depth examination of mutational scanning simulations and identification of binding energy hotspots for distinct nanobody classes. The review is focused on the analysis of mechanisms underlying synergistic binding of multivalent nanobodies that can be superior to single nanobodies and conventional nanobody cocktails in combating escape mutations by effectively leveraging binding avidity and allosteric cooperativity. We discuss how structural insights and protein engineering approaches together with computational biology tools can aid in the rational design of synergistic combinations that exhibit superior binding and neutralization characteristics owing to avidity-mediated mechanisms.

## 1. Introduction

Within the last two decades, two SARS-related coronaviruses (SARS-CoV) have crossed the species barrier to infect humans, including SARS coronaviruses 1 and 2 (SARS-CoV-1 and SARS-CoV-2) that caused the 2003 SARS epidemic and the current pandemic [1,2]. The coronavirus disease 2019 (COVID-19) pandemic has emerged as a global international health crisis that has spread over the world with far-reaching implications for the global economy, peace, and security [1,2]. The coronavirus SARS-CoV-2 is associated with the acute respiratory distress syndrome [1,2] and is similar to the severe acute respiratory syndrome (SARS) and Middle East respiratory syndrome (MERS) viruses [3]. The genomic sequences of the coronavirus SARS-CoV-2 revealed a high sequence similarity between the SARS-CoV-2, SARS and MERS proteins [4,5,6]. SARS-CoV-2 has four main structural proteins: spike (S) glycoprotein, small envelope (E) glycoprotein, membrane (M) glycoprotein, and nucleocapsid (N) protein, along with several accessory proteins [7,8,9]. Recent studies have identified that SARS-CoV-2 uses the angiotensin-converting enzyme 2 (ACE2) as host receptor [10,11,12]. SARS-CoV-2 infection is transmitted when the viral S glycoprotein binds to the ACE2, leading to the entry of S protein into host cells and followed by the fusion of the viral and cellular membranes mediated by the S2 subunit of the spike S protein [9,13,14]. The full-length SARS-CoV-2 S protein consists of two main domains, amino (N)-terminal S1 subunit and carboxyl (C)-terminal S2 subunit. The subunit S1 is involved in the interactions with the host receptor and includes an N-terminal domain (NTD), the receptor-binding domain (RBD), and two structurally conserved subdomains SD1 and SD2. The rapidly growing body of cryo-EM structures of the SARS-CoV-2 S proteins detailed distinct conformational arrangements of the S protein trimers in the prefusion form that are manifested by a dynamic equilibrium between the closed (“RBD-down”) and receptor-accessible open (“RBD-up”) form where the latter form is required for the S protein fusion to the viral membrane [15,16,17,18,19,20,21,22,23,24]. Protein engineering and structural studies showed that specific proline mutations can modulate stability of the SARS-CoV-2 S trimer [18] and lead to the accompanying thermodynamic shifts between the closed and open forms [19,20,21]. Dynamic structural changes that accompany SARS-CoV-2 S binding with the ACE2 host receptor were described in cryo-EM experiments showing a cascade of conformational transitions from a compact closed form weakened after furin cleavage to the partially open states and subsequently to the ACE2-bound open form [22]. The biophysical studies characterized conformational flexibility of the S protein trimers on the virion surface [23] showing that spontaneous conformational changes and population shifts between different functional states are salient features of spike dynamics in different biological environments, which is indicative of heterogeneous and adaptable conformational landscapes for the SARS-CoV-2 S trimers. Single-molecule Fluorescence (Förster) Resonance Energy Transfer (smFRET) studies of SARS-CoV-2 S trimer on virus particles revealed a sequence of conformational transitions from the closed state to the open state, suggesting that mechanisms of conformational selection and receptor-induced structural adaptation may both be in play acting synchronously [24].

The rapidly growing number of structural and biochemical studies of the SARS-CoV-2 S complexes with different classes of potent antibodies and antibody combinations have revealed multiple conformation-dependent epitopes, highlighting the link between conformational plasticity of SARS-CoV-2 S proteins and a remarkable adaptability and diversity of protein responses for eliciting specific binding and broad neutralization responses [25,26,27,28,29,30,31,32,33,34,35,36,37,38,39,40,41,42,43,44,45,46,47]. These studies have uncovered several prevalent mechanisms of SARS-CoV-2 S binding, showing that combinations of antibodies synergistically targeting distinct SARS-CoV-2 RBD epitopes can provide efficient cross-neutralization effects. Optimally designed antibody cocktails simultaneously targeting different binding epitopes on the SARS-CoV-2 RBD demonstrated also improved resilience against mutational escape [48,49,50,51,52,53].

SARS-CoV-2 S mutants with the enhanced infectivity profile have attracted an enormous attention in the scientific community following the evidence of the mutation enrichment via epidemiological surveillance, resulting in proliferation of experimental data and a considerable variety of the proposed mechanisms explaining functional observations. The emergence of variants of concern (VOC’s) with the enhanced transmissibility and infectivity profile including D614G variant [54,55,56,57], B.1.1.7 (alpha) [58,59,60,61], B.1.351 (beta) [62,63], B.1.1.28/P.1 (gamma) [64], and B.1.1.427/B.1.429 (epsilon) variants [65,66] revealed complex mechanisms underlying function and dynamics of the S proteins in different biological environments. Structural and biophysical studies characterized a diversity of S functional states for B.1.1.7 (B.1.1.7), B.1.351 (beta), P1 (gamma), and B.1.1.427/B.1.429 (epsilon) variants and showed that conformational plasticity of S proteins is modulated by mutations and determines the ability to evade host immunity and incur resistance to antibodies [67,68,69]. The recent VOC, omicron (B.1.1.529), displaying a large number of mutations in the S-RBD regions has further intensified the scientific and public interest and concerns about the role and mechanisms underlying the emergence of variants [70,71]. The latest structural investigation demonstrated that Omicron-B.1.1.529 mutational diversity can induce a widespread escape from neutralizing antibody responses [71].

## 2. Structural Biology of the SARS-CoV-2 S Protein Complexes with Nanobodies: Classification, Binding Epitopes, and Molecular Analyses

Although therapeutic antibodies attach to their targets with great affinity and specificity, they are limited in their capacity to reach tissue due to their large size and low stability. Nanobodies or single-domain antibodies provide important advantages over traditional antibodies, including their smaller size and robust biochemical properties such as high thermal stability, high solubility, and ability to be bioengineered into novel multivalent, multi-specific, and high-affinity molecules, making them a class of emerging powerful therapies against SARS-CoV-2 [72]. In the last decades, nanobodies have been actively exploited for diagnostics and therapy owing to their remarkable characteristics that favor their use over conventional antibodies. Nanobodies can also be readily produced recombinantly in bacteria, yeast, plants, and mammalian cell lines and be equipped with specific fluorescence, affinity, or epitope tags without losing their affinity or stability. The robustness and versatility of nanobodies in biophysical and therapeutic contexts have been important drivers of recent developments in nanobody engineering and numerous applications in biotechnology as powerful therapeutic and diagnostic agents. The ease of modification by genetic fusion, chemical or enzymatic conjugation makes nanobodies versatile tools for perturbation-based biophysical probing studies, microscopy, flow cytometry, mass cytometry, mass spectrometry, or non-invasive imaging methods. To sum up, the key advantages of nanobodies for the broad range of applications are: (a) the high thermal stability and robustness to extreme conditions; (b) penetrability properties cross the blood-brain barrier; (c) the ability to recognize novel epitopes that are not accessible to antibodies; (d) the improved bioavailability for pharmaceutical and biotechnology applications; (e) potential for bispecific antibody engineering; and (f) the ability to be expressed in both eukaryotic and prokaryotic systems [72,73,74,75].

**Figure 1:**
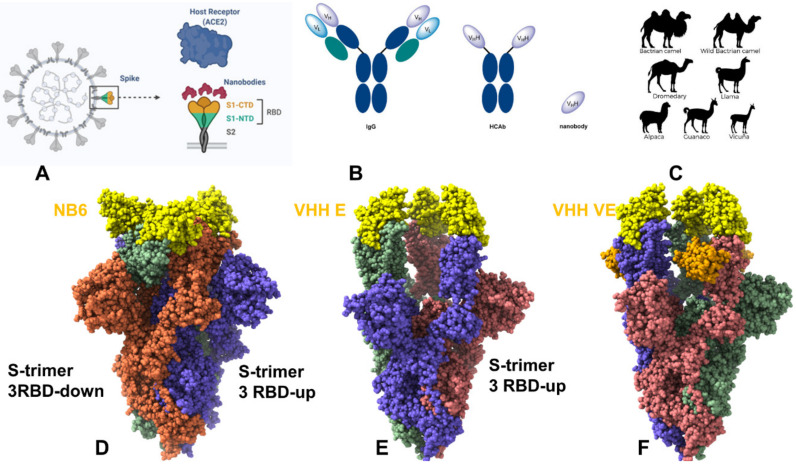
A schematic representation and comparison of conventional antibodies, heavy chain-only antibodies, and VHHs nanobodies (**A**,**B**). Adapted from https://app.biorender.com/biorender-templates (accessed on 3 February 2022). The antigen recognition site of a conventional antibody is formed jointly by the variable regions of the heavy (VH) and light (VL) chains. A nanobody corresponds to the variable region of a heavy chain only antibody. Immunization of camel, llama, alpaca as repertoire source to develop nanobodies (**C**) against interested targets allowed for a growing number of high-affinity nanobodies that effectively target the SARS-CoV-2 S proteins and induce effective neutralization. Cryo-EM structures of the SARS-CoV-2 S trimer in the complex with Nb6 nanobody, pdb id 7KKK. Nb6 nanobodies are shown in yellow (**D**). The SARS-CoV-2 S trimer in the complex with VHH E nanobody, pdb id 7KSG. VHH E is in yellow spheres (**E**). The SARS-CoV-2 S trimer in the complex with VHH V/VHH E nanobody, pdb id 7B18. VHH VE is in yellow/orange spheres. VHH E part in yellow and VHH V is in orange (**F**). The structures are shown in full spheres with protomers A,B,C colored in green, red, and blue. The rendering of SARS-CoV-2 S structures was done using the interactive visualization program UCSF ChimeraX.

There are currently nearly 500 available nanobodies targeting SARS-CoV-2 S protein by recognizing different binding sites in the S1 subunit as well as a smaller number of nanobodies interacting with S1 subunit regions [73,74,75,76,77,78]. Recent research efforts in the design, engineering, and structure-functional characterization of nanobodies and their binding with SARS-CoV-2 S proteins reflected a growing realization that nanobody combinations could deliver a powerful array of neutralizing and escape mutation resistant molecular assemblies capable of rationally exploiting distinct binding epitopes and the intrinsic plasticity of the SARS-CoV-2 S protein. Due to small sizes and thermal stability, nanobodies can bind in structurally different binding modes to often previously unappreciated epitopes with very high affinities, which is in contrast to antibody binding that often encounters steric hindrance when interacting with multiple epitopes [79,80]. Nanobodies also facilitate protein crystallization and are widely used in structural biology. Structural aspects and classification of the nanobodies binding with the SARS-CoV-2 S were recently discussed in a review [81], highlighting several classes of high affinity nanobodies (Figure 1).

**Figure 2:**
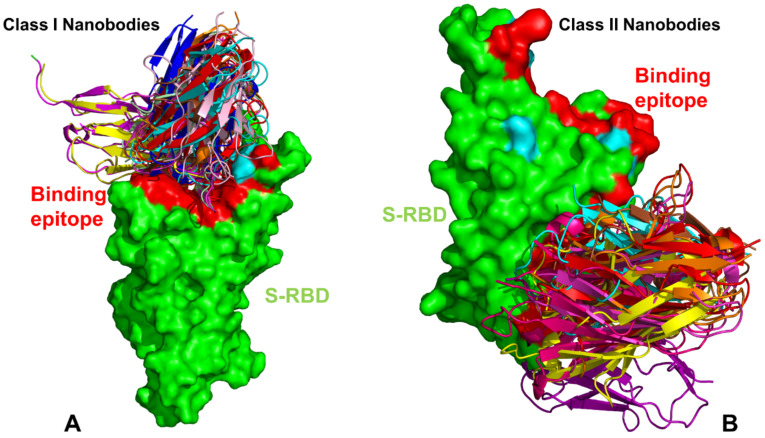
Structural classification and superposition of the SARS-CoV-2 S-RBD complexes with high-affinity nanobodies. (**A**) Structural superposition of the S-RBD complexes with class I nanobodies targeting the ACE2-binding region. The representative class I nanobodies shown are Nb6 (pdb id 7KKK), Nb20 (pdb id 7JVB), Sb45 (pdb id 7KLW), MR17 (pdb id 7C8W), SR4 (pdb id 7C8V), Sb14 (pdb id 7MFU), Sb16 (pdb id 7KGK), WNb2 (pdb id 7LX5), VHH-E (pdb id 7B14), C2 (pdb id 7OAO), H3 (pdb id 7OAP), NM1230 (pdb id 727), and Nanosota-1 (pdb id 7KM5). The S-RBD is shown in green surface. The superimposed nanobodies are shown in ribbons. A typical binding epitope for this class of nanobodies is shown in red-colored surface. The positions of K417, E484, and N501 are shown in cyan-colored surface on the RBD. For clarity, only E484 can be seen. (**B**) Structural superposition of the S-RBD complexes with class II nanobodies targeting the cryptic binding site in the core RBD region. The representative class II nanobodies shown included: C1 (pdb id 7OAP), F2 (pdb id 7OAY), Sb68 (pdb id 7KLW), VHH-U (pdb id 7KN5), VHH-V (pdb id 7KN6), VHH-W (pdb id 7KN7), NM1226 (pdb id 7NKT), WNb10 (pdb id 7LX5), and SR31 (pdb is 7D2Z). The S-RBD is shown in green surface. The superimposed nanobodies are shown in ribbons. Both ACE2-binding epitope and class II binding epitopes are shown in red-colored surface. The positions of K417, E484, and N501 are shown in cyan-colored surface on the RBD. The class II nanobodies target the S-RBD core region that is distinct from the ACE2- binding site.

**Figure 3:**
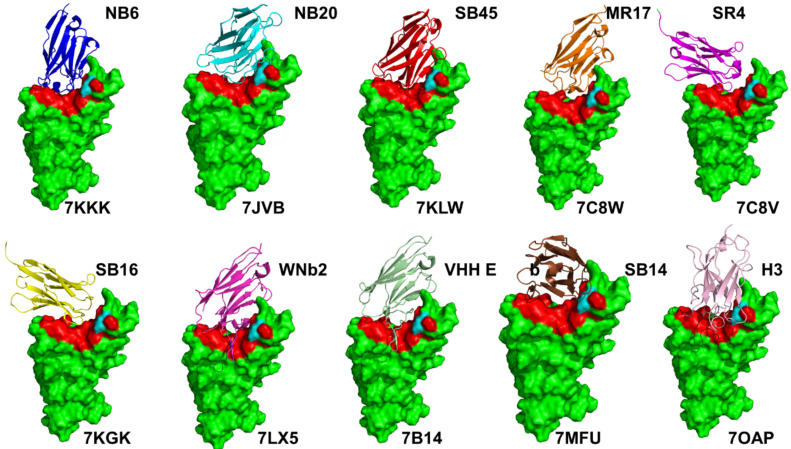
Structural details and binding modes for the class I nanobodies in complexes with S-RBD. The representative class I nanobodies shown are Nb6 (pdb id 7KKK), Nb20 (pdb id 7JVB), Sb45 (pdb id 7KLW), MR17 (pdb id 7C8W), SR4 (pdb id 7C8V) (top panel) and Sb16 (pdb id 7KGK), WNb2 (pdb id 7LX5), VHH-E (pdb id 7B14), Sb14 (pdb id 7MFU), and H3 (pdb id 7OAP) (bottom panel). The S-RBD is shown in green surface. The nanobodies are shown in ribbons. A typical binding epitope for this class is shown in red-colored surface. A high degree of similarity in binding modes can be seen for Nb6, Nb20, Sb45, MR17, WNb2, and VHH-E. A highly unusual binding mode was observed for SR4 and Sb16. The unique binding modes are seen for Sb14 and H3.

Here, we review in detail some of the recent structural and biophysical studies of high affinity nanobody complexes with SARS-CoV-2 S protein with a focus on classification, characterization of binding epitopes, and binding affinities and specificities. An ultra-potent synthetic nanobody Nb6 neutralizes SARS-CoV-2 by stabilizing the fully inactive down S conformation preventing binding with ACE2 receptor [82]. This study demonstrated that high-affinity trivalent nanobody mNb6-tri can simultaneously bind to all three RBDs and inhibit the interactions with the host receptor by locking the S protein in the inactive state [82]. The size-exclusion chromatography and mass spectrometry tools examined high-affinity RBD-targeting nanobodies that neutralize SARS-CoV-2 by binding to distinct and non-overlapping epitopes [83]. The revealed dominant epitope targeted by Nb20 and Nb21 nanobodies overlaps with the ACE2 binding site, showing that these nanobodies could competitively inhibit ACE2 binding [83]. The cryo-EM structures for different classes of nanobodies targeting novel epitopes suggested mechanisms of high-affinity and broadly neutralizing activity by exploiting epitopes that are shared with antibodies, but also include novel epitopes that are unique to the nanobodies [84]. High-resolution cryo-EM determination of SARS-CoV-1 S complexes with eight nanobodies discovered multiple potent neutralizing epitopes grouped into three clusters: cluster I (Nb20, N21) targets ACE2-binding sites and disrupts host receptor binding, cluster II (Nb35. Nb95, Nb105) targets highly conserved epitopes and retains activity against variants, and cluster III (Nb17, Nb360) recognizes unique epitopes that are inaccessible to antibodies [84]. This study provided the first systematic classification of ultrapotent nanobodies showing that class I nanobodies (Figure 2A and Figure 3) are the most potent neutralizing molecules preventing the virus from gaining entry to cells, while nanobodies can inactivate virus by targeting unique epitopes that prevent proper folding of S proteins and trap spike proteins in the unstable functionless conformation.

Highly synergistic nanobodies that target SARS-CoV-2 S protein and are resistant to escape were recently unveiled [85]. Using integrative structural modeling, this study provided a molecular perspective on the underlying mechanisms, showing how the nanobody repertoire can exhibit synergistic neutralizing activity by binding to proximal but non-overlapping epitopes (Figure 2), which improves potency while minimizing susceptibility to escape mutations [85]. Using human VH-phage library and protein engineering experiments, several unique VH binders were discovered that recognize two separate epitopes within the ACE2 binding interface with nanomolar affinity (Figure 3) [86]. This study also demonstrated that multivalent and bi-paratopic VH constructs can markedly increase the binding affinity and neutralization potency to the SARS-CoV-2 virus when compared to the individual VH domain [86]. Saturation mutagenesis of the S-RBD residues combined with fluorescence activated cell sorting for mutant screening identified escape mutants for five nanobodies and were mostly mapped to the periphery of the ACE2 binding site with K417, D420, Y421, F486, and Q493 emerging as notable escape hotspots [87].

**Figure 4:**
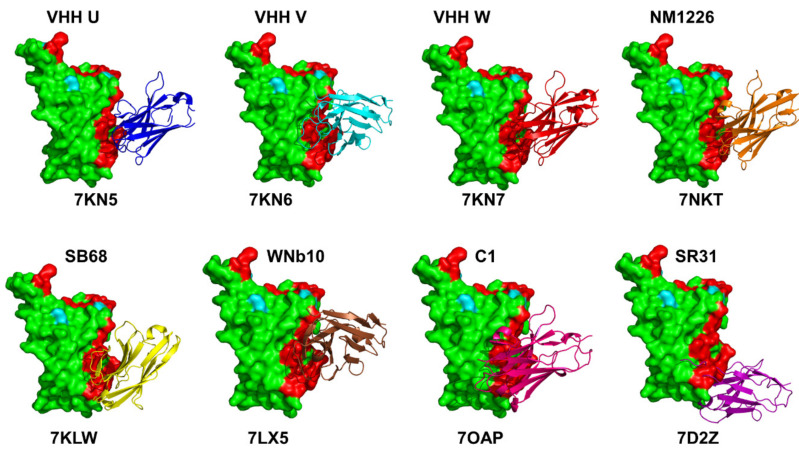
Structural details and binding modes for the class II nanobodies in complexes with S-RBD. The structures are shown for VHH-U (pdb id 7KN5), VHH-V (pdb id 7KN6), VHH-W (pdb id 7KN7), NM1226 (pdb id 7NKT) (top panel) and Sb68 (pdb id 7KLW), WNb10 (pdb id 7LX5), C1 (pdb id 7OAP), and SR31 (pdb id 7D2Z) (bottom panel). The S-RBD is shown in green surface. The nanobodies are shown in ribbons. Both ACE2- binding epitope and class II binding epitopes are shown in red-colored surface. A similarity of binding modes is seen for VHH U, VHH W, and NM1226. The unique binding modes in the cryptic binding site are seen for Sb68, WNb10, and especially SR31 nanobody.

A wide range of rationally engineered nanobodies with efficient neutralizing capacity and resilience against mutational escape was recently unveiled, including the llama-derived nanobody VHH E bound to the ACE2- binding epitope and three alpaca-derived nanobodies VHH U, VHH V, and VHH W that bind to a different cryptic RBD epitope (Figure 2B and Figure 4) [88]. Two types of nanobody fusions were engineered in this study: multivalent nanobodies that target the ACE2 binding site and bi-paratopic fusions of two nanobodies that target distinct binding interfaces and amplify neutralization by activation of the S protein. The engineered multivalent nanobodies displayed high avidity towards the spike protein and were 100-fold more potent than their monomeric nanobodies, while bi-paratopic nanobody fusion (VHH EV and VHH VE) prevented emergence of escape mutants that reduced efficiency of the binary nanobody cocktails E + U, E + V and E + W [88].

Using single-domain antibody library and PCR-based maturation, two closely related and highly potent nanobodies, H11-D4 and H11-H4, were reported [89]. The crystal structures of these nanobodies bound to the S-RBD revealed binding to the same epitope, which partly overlaps with the ACE2 binding surface. X-ray crystallography, cryo-EM study, and epitope binning experiments identified a number of high-affinity nanobodies and nanobody cocktails that block ACE2 binding [90]. All tested nanobodies bind with dissociation constant *K*_D_ ranging from 0.14 to 19.49 nM, with high antibody affinities (*K*_D_ < 0.5 nM) shown for the top four nanobodies WNb2, WNb7, WNb15, and WNb36. Neutralizing activity for ten nanobodies (WNb67, 41, 36, 2, 53, 7, 50, 15, 10, and 19) were at ≤80 nM. Some of the most potent nanobodies included WNb2 that binds to the ACE2-binding site (Figure 3) and WNb10 nanobody that contacts RBD residues N437, V503, and Y508 and the second site that involves RBD residues 368–377 (Figure 4). This study showed WNb2 can also bind to E484A or E484K with relative EC_50_ values of 2 and 5 nM, respectively, and competitively inhibited E484K interaction with ACE2 [90].

Using alpaca immune libraries against the SARS-CoV-2 S-RB protein, 45 infection-blocking VHH nanobodies were generated and the most potent molecules neutralized SARS-CoV-2 at 17–50 pM concentration binding to the open and closed states of the S protein as revealed in the X-ray and cryo-EM structures [91]. The engineered nanobody tandems and nanobody monomers showed very high affinity and bound synergistically to the opposite sides of the RBD. Moreover, these nanobodies can still block RBD-ACE2 interactions for a combination of escape mutations (K417T, E484K, N501Y, L452R) and neutralize B.1.351 variant at low-picomolar concentrations. The cryo-EM structures of the tandem fusion of the Re5D06 nanobody binding to the RBM region and hyper-thermostable Re9F06 nanobody that binds the RBD core epitope revealed a molecular basis for synergistic binding with resistance to immune-escape mutants [91]. This seminal biophysical investigation highlighted the importance of nanobody tandems in which one of the nanobodies targets the cryptic epitope (class II). These nanobodies can be effective in compensating for losses due to escape mutants in the ACE2 binding epitope as structural and functional constraints tend to prevent emergence of escape mutations in the RBD core region. This cryptic epitope (Figure 4) could be under lower escape pressure and VHHs against this epitope would likely tolerate future escape mutations better than the class I epitope nanobody binders [91].

The recent discovery of nanobodies and nanobody cocktails from camelid mice and llamas that neutralize SARS-CoV-2 variants showed the remarkable ability of multivalent nanobodies to combat escaping mutations through synchronized avidity between binding epitopes [92]. This study described a highly potent group of llama nanobodies Nb17, Nb19, and Nb56 directly targeting the ACE2-binding interface (Figure 3) and picomolar nanobodies Nb12 and Nb30 binding to a conserved RBD epitope outside of the ACE2-binding motif, which is not accessible to human antibodies (Figure 4) [92]. SARS-CoV-2 RBD-specific nanobodies with high stability were identified using phage display and the fusion of two nanobodies with non-overlapping epitopes resulted in hetero-bivalent nanobodies with significantly higher RBD binding affinities (*K*_D_ of 59.2 pM and 0.25 nM) and greatly enhanced SARS-CoV-2 neutralizing potency [93]. The X-ray crystal structures of sybodies (synthetic nanobodies) that effectively inhibit the ACE2 binding interaction and neutralize viral infectivity were reported and a bispecific construct, Sb15-Sb68 blocked ACE2 binding (Figure 3 and Figure 4) [94]. A bispecific monomeric nanobody that induces SARS-CoV02 S trimer dimers and neutralizes SARS-CoV-2 in vivo was reported [95]. The engineered Fu2 nanobody interacts simultaneously with two RBDs from different SARS-CoV-2 S trimers inducing the highly unusual mode of binding and formation of spike dimers, leading to potent neutralization of the virus as well as the B.1.351 and B.1.617.2 variants. Remarkably, not only does the F2 nanobody act by targeting two epitopes, but it also locks in the S trimers in very constrained conformations that completely block spike function [95].

The nanobodies C5, H3, C1, F2 engineered as homotrimers with picomolar affinity for the S-RBD were described structurally, showing that C5 and H3 overlap the ACE2 epitope, while C1 and F2 bind to a different epitope [96]. Consistent with other recent reports, nanobodies that bound to both sites showed very potent neutralization activity as multivalent trimers, with the C5 trimer demonstrating complete inhibition of infection at < 100 pM in a PRNT assay [96]. The development and characterization of a series of nanobody candidates that specifically target the SARS-CoV-2 RBD using screening of the nanobody phage display library, and two rounds of affinity maturation and structural determination were reported, revealing the crystal structure of SARS-CoV-2 RBD complexed with Nanosota-1 nanobody that binds to the SARS-CoV-2 RBD and completely blocks the binding of ACE2 [97]. A rapid selection of 99 sybodies against RBD by in vitro selection using three libraries produced six neutralizing sybodies, SR4, MR3, MR4, MR17, LR1, and LR5 that bind to S-RBD with high affinity and display high neutralization activity against SARS-CoV-2 pseudoviruses [98]. Despite targeting the ACE2 binding region, these sybodies can assume completely different binding modes and interaction patterns.

These studies suggested that nanobody mixtures and rationally engineered bi-paratopic nanobody constructs could offer a promising alternative to conventional monoclonal antibodies and may be advantageous for controlling a broad range of infectious variants, while also suppressing the emergence of virus escape mutations. Furthermore, bi-paratopic nanobodies showed significant advantages compared to monoclonal antibodies, single nanobodies, and nanobody cocktails by effectively leveraging binding avidity and allosteric cooperativity mechanisms in combating escape mutations.

## 3. Biophysical Characterization and Protein Engineering of SARS-CoV-2 Complexes with Nanobodies: From Monomeric to Multivalent Formats and Synergistic Combinations in the Quest for High-Affinity and Mutation-Resistant Nanobodies

Biochemical and biophysical tools together with novel protein engineering approaches have been instrumental in the design of nanobodies and suggested that multivalent protein nanotechnology and chemistry can lead to rational synergistic combinations that exhibit superior binding and neutralization characteristics owing to avidity-mediated mechanisms [99]. Linking nanobodies via flexible linkers to create bivalent and trivalent assemblies can significantly increase binding due to avidity [99]. A tour-de-force in-depth analysis of the SARS-CoV-2 S nanobodies described a large repertoire of strongly neutralizing and escape resistant nanobodies with a total of 71 nanobodies bound to the S-RBD regions, 19 targeting non-RBD regions of S1 subunit and 26 binding to the S2 subunit [85]. Surface plasmon resonance (SPR) quantified the kinetic properties and high binding affinities of the selected nanobodies with >60% binding with *K*_D_ < 1 nM, and two with single-digit picomolar affinities. Two strategies were employed to ensure the generation of nanobodies whose binding is resistant to emerging variants. A large diversity of high quality RBD nanobodies was generated to maximize the probability of identifying ones that are refractory to escape as well as design of nanobodies targeting the non-RBD regions. The nanobodies (S1-1, S1-6, S1-RBD-9, S1-RBD-11, S1-RBD-15, and S1-RBD-35) inhibited B.1.1.7/20I/501Y.V1 and B.1.351/20H/501Y.V2 variants with high affinity [85]. A combination of S1-23 and S1-1 nanobodies, which bind to opposite sides of the RBD, dramatically increased the potency of both nanobodies by ~32- and ~21-fold, respectively. A group of resistant mutations in the dynamic RBM region (F490S, E484K, Q493K, F490L, F486S, F486L, and Y508H) was shown to evade many individual nanobodies [85]. In a conceptually similar approach, synthetic non-overlapping SARS-CoV-2 nanobodies were assembled into bi-paratopic and multivalent formats with linkers showing ~600 and 1400-fold increase the binding affinity and virus neutralization potency, respectively [86].

A series of high affinity sybodies Sb14, Sb16, Sb45, Sb68, featured binding to the immobilized RBD with *K*_D_ values of 6.8 to 62.7 nM [94]. The ternary structures of Sb45–RBD–Sb68 and Sb14–RBD–Sb68 showed that the bivalent and multivalent binding to non-overlapping RBD sites can increase neutralization potential through synergistic avidity of complementary nanobodies against the virus [94]. In another study, trivalent versions of the four nanobodies were constructed by joining the VHH domains with a glycine-serine flexible linker [96]. The nanobody homo-trimers (C5, C1 and H3) were produced by transient expression and binding of the trimeric nanobodies to the DS-RBD was measured by SPR revealing 10- to 100-fold enhancement in *K*_D_ binding constant as compared to the monomers [96]. The proposed mechanism of resistance to mutational escape by nanobody combinations may arise from reduction in the number of allowed substitutions to confer resistance and the elevated genetic barrier for escape. Hence, mixtures or multimeric versions of high-affinity nanobodies can represent a robust strategy for design of escape-resistant therapeutics.

Six high affinity neutralizing sybodies, SR4, MR3, MR4, MR17, LR1, and LR5, were described featuring a range of dissociation constant *K*_D_ from 83.7 nM for MR17 to 1.0 nM for MR3 [98]. Sybody engineering using the bi-paratopic fusion of two different sybodies, the Fc-fusion and tandem fusion of the same sybody, increases the binding affinity and neutralizing activity [98]. The bi-paratopic LR5-MR3 sybody derivative was more potent than either LR5 or MR3 sybody alone, and LR5-MR3 is also more tolerant to escape mutants due to the ability to recognize two distinct epitopes [98]. Potential avidity effects of Sb14, Sb16, Sb45, Sb68 sybodies were also investigated by generating genetically fused sybody constructs to human IgG1 Fc domains [100]. The respective sybody-Fc constructs (Sb14-Fc, Sb15-Fc, Sb16-Fc, Sb45-Fc, and Sb68-Fc) exhibited IC_50_ values in the range of 8 to 50 nM, considerably improving the respective values of the individual sybodies which ranged from 138 to 1250 nM [100].

Different constructs consisting of Sb15 and Sb68 fused via a flexible linker (GGGGS) of various lengths resulted in bispecific sybodies with dramatically improved neutralization activity as compared to the single binders. The structural and binding data confirmed that Sb15 and Sb68 bind to non-overlapping epitopes on the RBD surface and exhibit synergistic neutralizing effects [100]. A series of 11 unique nanobodies was revealed in another study that effectively block the interaction of the ACE2 to isolated RBD, S1 domain, and homotrimeric S protein [101]. By testing combinations, substantially improved IC_50_ values were obtained, indicating a highly synergistic effect of combinations targeting simultaneously different epitopes within the RBD. The IC_50_ values were obtained for the most potent inhibitory nanobodies NM1228 (0.5 nM), NM1226 (0.85 nM), and NM1230 (2.12 nM), and NM1226 and NM1228, showed the highest neutralization potency with IC_50_ values of ~15 and ~7 nM [101]. The most potent inhibitory and neutralizing candidates NM1226, NM1228, and NM1230 were tested in the multiplex binding assay and for viral neutralization, showing that the combination of NM1226 and NM1230 led to an increased effect in competing with ACE2 binding to RBD, which is two- or five-fold lower compared to treatment with individual NM1226 or NM1230, respectively.

A panel of 37 anti-SARS-CoV-2 spike nanobodies was characterized as both monovalent and IgG Fc-fused bivalent modalities [102]. The analysis of binding characteristics showed that both monovalent and bivalent nanobodies have high affinity and high cross-reactivity against spike variants. The reformatting of monovalent nanobodies to bivalent nanobodies caused a significant improvement in neutralizing efficacy and was highest for anti-RBD nanobodies (2–100-fold). A total of 12 out of 20 tested bivalent nanobodies exhibited high efficacy in neutralizing the Delta variant. Of these nanobodies, nine were capable of neutralizing all tested variants [102] For the first time, this study demonstrated high epitope diversity, revealing that several NTD- and S2-specific nanobodies were potent and efficacious neutralizers in vitro and in vivo as well as remaining potent against the Alpha, Beta, Gamma, Delta, and Kappa variants [102]. Using computer-aided design, multi-specific VHH antibodies fused to human IgG1 Fc domains were constructed based on the epitope predictions for leading VHHs [103]. The tri-specific, bi-specific, and mono-specific VHH-Fcs were tested for their ability in vitro for SARS-CoV-2 S1 protein binding and S/ACE2 blocking, showing higher binding for the tri-specific VHH-Fcs 3F-1B-2A (*K*_D_~0.047 nM) and 1B-3F-2A (*K*_D_~0.095 nM) than that of individual component VHH-Fcs or in combination. The tri-specific antibodies are advantageous as therapeutic agents because they simultaneously bind multiple epitopes within the S1 protein RBD that increase their antigen-binding affinity and avidity. The high-affinity nanobodies that neutralize SARS-CoV-2 by binding to distinct and non-overlapping epitopes [83] with pico- to femtomolar affinities were further engineered to produce linker-based homo-, heterodimeric, and homotrimeric nanobodies. The resulting multivalent nanobodies showed exceptional avidity and ultrahigh neutralization potency against SARS-CoV-2 virus. In particular, an ultrapotent homotrimeric construct, Pittsburgh inhalable Nanobody 21 (PiN-21), efficiently blocked SARS-CoV-2 infectivity [83]. Using Syrian hamsters that model moderate to severe COVID-19 disease, a subsequent study demonstrated the high efficacy of PiN-21 to prevent and treat SARS-CoV-2 infection [104].

Many other recent studies have demonstrated and confirmed the potential of linker-based bi-paratopic or multi-paratopic nanobodies for enhanced blocking and neutralization of SARS-CoV-2 variants. The avidity effect was also observed by Hanke and colleagues that presented a bispecific monomeric nanobody that can promote the formation of the S trimer dimers and neutralizes SARS-CoV-2 in vivo [95]. This study demonstrated that multimerization of nanobodies can substantially improve virus neutralization potency, and the cryo-EM structure of the Fu2-spike complex with S protein revealed a remarkable head-to-head dimerization of the trimeric spike, bound by six molecules of Fu2 [95]. Another strategy to increase sybody potency by bi-paratopic fusion was proposed [105] using a sybody SR31 that binds RBD with high affinity, but does not perturb ACE2 binding. A bi-paratopic fusion MR17-SR31 displayed a remarkable increase in binding affinity *K*_D_ of 0.3 nM that was lower than MR17 (*K*_D_ = 83.7 nM) by 230 fold and lower than SR31 (*K*_D_ = 5.6 nM) by 17 fold. Compared with mono-paratopic divalent nanobodies or monoclonal antibodies, bi-paratopic nanobodies are more resistant to escape mutants because simultaneous mutations at two distinct and relatively remote epitopes should occur at a much lower rate than at a single epitope [105]. A multivalent engineering approach of potent Nb91-hFc and Nb3-hFc nanobodies was employed to improve the neutralizing capacity [106]. The neutralizing ability of triNb91-hFc (IC50 = 4.89 nM) improved 11.06 fold compared to the monovalent form (IC50 = 54.07 nM), and triNb3- hFc (IC50 = 4.70 nM) improved 6.88 fold than monovalent construct (IC50 = 32.36 nM) by the pseudo-virus luciferase assay [106]. The heterodimer nanobody Nb91-Nb3-hFc exhibited the highest neutralizing ability, with an IC50 at 1.54 nM against pseudo-typed SARS-CoV-2. Multivalent engineering approach was also used to achieve large synergistic improvements in the neutralizing activity of a SARS-CoV-2 cross-reactive nanobody (VHH-72) initially generated against SARS-CoV [107]. A hexavalent VHH-72 nanobody retains binding to spike proteins from multiple highly transmissible SARS-CoV-2 B.1.1.7 and B.1.351 variants.

Avidity-inspired nanobody therapeutics can leverage the emerging evidence how binding affinity, avidity and cooperativity are balanced in a complex thermodynamic mechanism of synchronous binding of multivalent nanobody constructs [108]. The recent biophysical studies indicated that avidity-driven mechanisms may underlie functional effects of nanobody combinations and multivalent nanobody constructs to prevent viral escape, making it possible to rationally engineer the desirable level of binding specificity and generation of ultra-potent molecules for targeting SARS- CoV-2 S proteins. In contrast to the binding affinity, which can be accurately determined by considering the relationship between the microscopic association and dissociation rates, the avidity is a phenomenological macroscopic characteristic that linked not only to the binding strength, but also degree of cooperativity, the valency of the interaction partners, structural rearrangements of proteins, and diffusional properties [109]. A potentially promising strategy may involve multivalent nanobody engineering coupled with *de novo* protein design to generate complex non-immunogenic protein scaffolds for use in nanobodies [110]. This pioneering work offered a general approach for forming precisely oriented antibody assemblies with controlled valency by uniting topology, geometry, and function requirements for the computational design of two-component nanocages. The biological phenomenon that is central to nanobody engineering is based on a protein self-assembly mechanism in which a single building block is often sufficient to create structures with complex and predetermined shapes and topologies that can enable multivalent binding, ultra- sensitive regulation, and compartmentalization in cellular environments.

## 4. Computational Studies of SARS-CoV-2 S Protein Binding Mechanisms: Structure, Dynamics, and Allostery

Computer simulations and protein modeling played an important role in shaping up our understanding of the dynamics and function of SARS-CoV-2 glycoproteins [111,112,113,114,115,116,117,118,119,120]. All-atom MD simulations of the full-length SARS-CoV-2 S glycoprotein embedded in the viral membrane with a complete glycosylation profile were first reported by Amaro and colleagues, providing the unprecedented level of details and significant structural insights about functional S conformations [113]. A “bottom-up” coarse-grained (CG) model of the SARS-CoV-2 virion integrated data from cryo-EM, X-ray crystallography, and computational predictions to build molecular models of structural SARS-CoV-2 proteins assemble a complete virion model [114]. By establishing the blueprint for computational modeling, these studies paved the way for simulation-driven studies of SARS-CoV-2 spike proteins, also showing that conformational plasticity and the alterations of the SARS-CoV-2 spike glycosylation can synergistically modulate complex phenotypic responses to the host receptor and antibodies. Multi-microsecond MD simulations of a 4.1 million atom system on a viral membrane with four full-length, fully glycosylated, and palmitoylated S proteins provided another fundamental milestone in the building foundation for a simulation-driven modeling of SARS-CoV-2 S proteins [115]. This study described a comprehensive mapping of generic antibody binding signatures and provide a detailed atomistic characterization of the antibody and vaccine epitopes. MD simulations also revealed a balance of hydrophobic interactions and elaborate hydrogen-bonding network in the SARS-CoV-2-RBD interface [121]. A critical analysis of computer simulation studies of SARS-CoV-2 S proteins provided an insightful critical assessment of existing approaches and identified gaps between the experiments and atomistic simulations advocating for a community-based effort to build the infrastructure and foundation for large-scale atomistic modeling of SARS-CoV-2 structural proteins and broad adaptation of mesoscale simulations of the complete virion [122].

More recent extensive simulation studies and free energy landscape mapping studies of the SARS-CoV-2 S proteins and mutants detailed conformational changes and diversity of ensembles, demonstrating enhanced functional and structural plasticity of S proteins [123,124,125,126,127,128,129]. Using data analysis and protein structure network modeling of MD simulations, residues that exhibit long-distance coupling with the RBD opening, including sites harboring functional mutations D614G and A570D, which points to the important role of D614G variant in modulating allosteric communications in the S protein [125]. The free energy landscapes of the S protein and modeling of the RBD opening using the nudged elastic pathway optimization revealed a cryptic allosteric pocket located near the D614 hinge position [126]. Using computational modeling, it was shown that the D614G mutation may affect the inter-protomer energetics between S1 and S2 subunits that promote the formation of the open S protein form [127].

Several computational studies examined the effects of global circulating mutations on dynamics and stability of the SARS-CoV-2 S proteins. MD simulations probed the effects of the D614G mutation, suggesting that the variant favors an open conformation in which S-G614 protein maintains the symmetry in the number of persistent contacts between the three protomers [128]. Markov modeling characterized the dynamics of the S protein and mutational variants, predicting the increase in the open state occupancy for the D614G mutation due to the increased flexibility of the closed state and the enhanced stabilization of the open form [129]. The energy analysis of the S-D614 and S-G614 proteins in the closed and partially open conformations showed that local interactions near D614 position may be energetically frustrated and become stabilized in the S-G614 mutant through strengthening of the inter-protomer association between S1 and S2 regions [130].

Computational and biophysical kinetics studies of the SARS-CoV-2 S trimer interactions with ACE2 using the recent crystal structures also provided important insights into the key determinants of the binding affinity and selectivity [118,131,132,133]. Microsecond all-atom MD simulations of the SARS-CoV-2 S-RBD complex with ACE2 in the absence and presence of external force examined the effects of alanine substitutions and charge reversal mutations of the RBD residues, showing that the hydrophobic end of RBD serves as the main energetic hotspot for ACE2 binding [118]. In a series of studies, we suggested that the SARS-CoV-2 spike protein can function as an allosteric regulatory engine that fluctuates between dynamically distinct functional states [134,135]. By combining coarse-grained and atomistic MD simulations with coevolutionary analysis and network modeling, our recent investigations presented evidence that SARS-CoV-2 S proteins are functionally adaptable and allosterically regulated machines that exploit the intrinsic plasticity of functional regions and versatility of allosteric hotspots to modulate complex phenotypic responses to the host receptor and antibodies [136,137,138]. Computational modeling of the SARS-CoV-2 S trimer complexes with H014, S309, S2M11, and S2E12 antibodies showed that binding can differentially modulate the allosteric potential of spike residues and exert control over long-range communications [137]. The results suggested that antibody-escaping mutations can target allosteric switch centers involved in regulation of global motions and long-range interactions. Subsequent studies of the SARS-CoV-2 S trimer binding with different classes of antibodies revealed that dynamically adaptable allosteric hotspots of the S proteins coincided with the functional sites targeted by global circulating mutations [138]. Through functional dynamics analysis and perturbation-response scanning of the SARS-CoV-2 spike protein residues in the unbound form and antibody-bound forms, we examined how antibody binding can modulate allosteric propensities of spike protein residues and determine allosteric hotspots that control signal transmission and global conformational changes [139]. These results showed that residues K417, E484, and N501 targeted by circulating mutations correspond to a group of versatile allosteric centers in which small perturbations can modulate collective motions, alter the global allosteric response, and elicit binding resistance. This study suggested that SARS-CoV-2 S protein may exploit plasticity of specific allosteric hotspots to generate escape mutants that alter response to antibody binding without compromising activity of the spike protein [139]. A computational approach for rapid mutational scanning of binding energetics and residue interaction networks in the SARS-CoV-2 spike protein complexes was recently proposed [140]. Using this approach, we integrated atomistic simulations and conformational landscaping of the SARS-CoV-2 spike protein complexes with the ensemble-based mutational screening and network modeling to characterize mechanisms of structure-functional mimicry and resilience to mutational escape by the ACE2 protein decoy and de novo designed miniprotein inhibitors [140]. Collectively, our computational studies offered evidence that the SARS-CoV-2 spike protein can function as an allosterically regulated machine that exploits plasticity of allosteric hotspots to fine-tune response to antibody binding [134,135,136,137,138,139,140,141,142]. These studies showed that examining allosteric behavior of the SARS-CoV-2 S proteins may help to uncover functional mechanisms and rationalize the diverse experimental data.

Using MD simulations and in silico mutational profiling of protein stability and binding, we recently examined dynamics and energetics of the SARS-CoV-2 complexes with single nanobodies Nb6 and Nb20, VHH E, a pair combination VHH E+U, a bi-paratopic nanobody VHH VE, and a combination of CC12.3 antibody and VHH V/W nanobodies [143]. This study characterized the binding energy hotspots in the SARS-CoV-2 protein and complexes with nanobodies providing a quantitative analysis of the effects of circulating variants and escaping mutations on binding that is consistent with a broad range of biochemical experiments. The results suggested that mutational escape may be controlled through structurally adaptable binding hotspots in the receptor-accessible binding epitope that are dynamically coupled to the stability centers in the distant binding epitope targeted by VHH U/V/W nanobodies [143]. Using computer-based design of protein–protein interactions, a number of nanobodies were engineered in silico and selected based on the free energy landscape of protein docking verified by the recently reported cocrystal structures [144]. The predicted stabilities and high binding affinities of the 11 designed nanobodies indicated the potential of the developed computational framework to design effective agents to block the infection of SARS-CoV-2.

Another computational study examined binding mechanisms of neutralizing nanobodies targeting SARS-CoV-2 S proteins [145]. All-atom MD simulations totaling 27.6 μs in length using the recently solved structures of the RBD of SARS-CoV-2 S protein in complex with nanobodies H11-H4 [145], H11-D4, and Ty1 revealed interactions between RBD and the nanobodies and estimated that the binding strength of the nanobodies to RBD is similar to that of ACE2 [145]. Together, our MD simulations suggest that H11-H4 and Ty1 abrogate ACE2 binding via different mechanisms, whereas H11-D4 is the least effective inhibitor among the three examined nanobodies, which is consistent with the experimental studies showing the lowest *K*_D_ value for Ty1 and the highest for H11-D4 nanobody [89,95].

## 5. Exploring Computational Approaches for Mutational Profiling of SARS-CoV-2 S Protein Binding with Nanobodies and Characterization of the Binding Energy Hotspots

The mutational heatmaps provided a visual representation of screening, pointing to the S-RBD residues with high sensitivity to modifications, and therefore, considered as potential binding energy hotspots [143]. Using ensemble-based mutational profiling of stability and binding affinities, we identified critical hotspots and characterized molecular mechanisms of SARS-CoV-2 S binding with single nanobodies, nanobody cocktails, and bi-paratopic nanobodies. Here, we present the results of mutational scanning for two major classes of nanobodies that were initially discussed in [143] and are further expanded in the current study. In silico mutational scanning is based on the equilibrium ensembles of conformations obtained from MD simulations to systematically profile the S-RBD residues and compute the binding free energy changes caused by amino acid modifications for studied SARS-CoV-2 S-RBD complexes with nanobodies.

**Figure 5:**
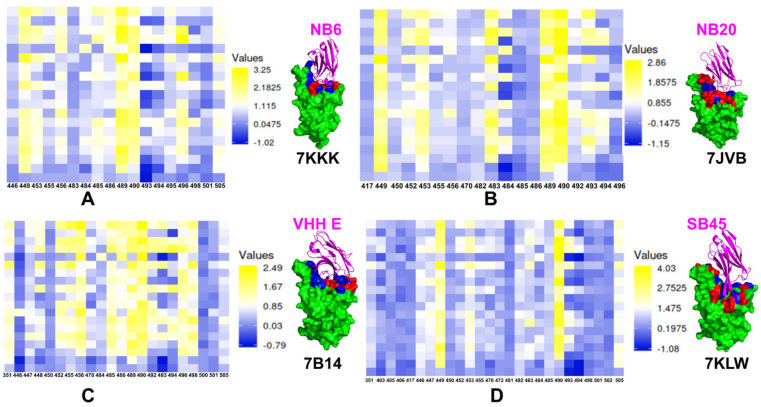
Mutational profiling of the SARS-CoV-2 S binding with class I nanobodies. (**A**) The mutational scanning heatmap for the SARS-CoV-2 S complex with Nb6 nanobody and structural map of the S-RBD binding epitope in the complex with Nb6. The S-RBD is shown in green surface. The epitope residues are shown in red and the binding energy hotspots from are shown in blue surface. (**B**) The mutational scanning heatmap and structural map for the SARS-CoV-2 S complex with Nb20 nanobody. The annotations for the epitope residues and the binding energy hotspots are as in panel A. (**C**) The mutational scanning heatmap and structural map for the SARS-CoV-2 S complex with VHH E nanobody. The annotations for the epitope residues and the binding energy hotspots are as in panel A. (**D**) The mutational scanning heatmap and structural map for the SARS-CoV-2 S complex with Sb45 nanobody. The annotations for the epitope residues and the binding energy hotspots are as in panel A. The heatmaps show the computed binding free energy changes for 19 single mutations on the binding epitope sites. The squares on the heatmap are colored using a 3-colored scale from blue to yellow with yellow indicating the largest unfavorable effect on binding.

For the class I of nanobodies that target the ACE2-binding site region, we compared the mutational heatmaps for Nb6 (pdb id 7KKK), Nb20 (pdb id 7JVB), VHH E (pdb id 7B14), and Sb45 (pdb id 7KLW) (Figure 5). The observed binding hotspots for Nb6 corresponded to the hydrophobic RBD residues Y449, L453, L455, F456, G485, Y489, F490, G496, and Y505 that were confirmed in deep mutagenesis scanning experiments as important functional positions for the ACE2 binding (Figure 5A). Mutational scanning analysis correctly reproduced the experimentally known sensitivity of sites targeted by common resistant mutations of many individual nanobodies (F490S, E484K, Q493K, F490L, F486S, F486L, and Y508H) [85]. Computational mutational profiling suggested several commonly shared hotspots for Nb6 and Nb20 nanobodies that included Y449, F456, Y489, and F490. The nanobody-specific binding hotspots for Nb20 were aligned with V483, Y489, F490, L492, and Q493 residues (Figure 5B). Consistent with the experimental data, mutational scanning of VHH E nanobody binding to the SARS-CoV-2 S protein revealed a large number of binding hotspots including L452, L455, F456, G485, F486, Y489, F490, Q493, G496, and Q498 (Figure 5C). Sb45 uses both long CDR2 and CDR3 loops along both sides of the RBD surface [94], while VHH E uses a long CDR3 loop engaging only one side of the RBD surface [88].

These results strongly support the notion that that functional and structural plasticity can be important to mechanisms by which mutations can alter the antigenic responses and elicit antibody evasion. Indeed, we found that the sites of mutational escape to nanobodies corresponded to flexible energetic centers that are involved in the network of local binding interactions and execution of functional cooperative movements in response to binding. As a result, mutations in these positions could have strong cumulative effects by compromising nanobody recognition and binding affinity as well as affecting functional dynamics and protein response to perturbations [143].

Analysis of the buried surface area (interfaces between RBD and ACE2 or the sybodies) showed that Sb45 cover more surface area than ACE2 and other known sybody–RBD complexes [94]. This is reflected in the larger epitope for Sb45 that recruits a number of RBD residues as binding energy hotspots, including G446, G447, Y449, L452, Y453,V483, F490, Q493 (Figure 5D). In general, binding energy hotpots shared by the nanobodies Nb6, Nb20, VHH E, Sb45 include Y449, L452, Y489, and F490. Although the RBD epitope for Sb45 is larger, the number of prominent binding hotspots is greater for the VHH E nanobody, which may partly explain a more favorable binding affinity for VHH E [88].

**Figure 6:**
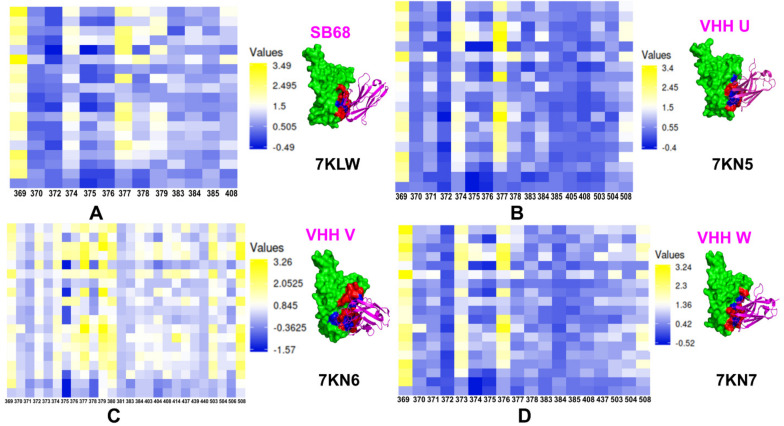
Mutational profiling of the SARS-CoV-2 S binding with class I nanobodies. (**A**) The mutational scanning heatmap and structural map for the SARS-CoV-2 S complex with Sb68 nanobody. The S-RBD is shown in green surface. The epitope residues are shown in red and the binding energy hotspots from are shown in blue surface. (**B**) The mutational scanning heatmap and structural map for the SARS-CoV-2 S complex with VHH U nanobody. The annotations for the epitope residues and the binding energy hotspots are as in panels A,B. (**C**) The mutational scanning heatmap and structural map for the SARS-CoV-2 S complex with VHH V nanobody. (**D**) The mutational scanning heatmap and structural map for the SARS-CoV-2 S complex with VHH W nanobody. The heatmaps show the computed binding free energy changes for 19 single mutations on the binding epitope sites. The squares on the heatmap are colored using a 3-colored scale from blue to yellow with yellow indicating the largest unfavorable effect on binding.

The mutational scanning analysis of the class II nanobodies binding to the RBD core site located away from the ACE2 interface demonstrated a consistent pattern of hotspots (Figure 6). The binding hotspots for Sb68 corresponded to stable residues Y369 and F377. This is consistent with the mode of binding for VHH U showing important interacting positions in Y369, S371, F374, F377, K378, and C379 with most prominent hotspots Y369 and F377 (Figure 6A). The binding interface for Sb68 is fairly small and structural analysis showed that Sb68 is not fully optimal for the binding cleft. The experimentally observed binding affinity of ~40 nM is lower than that of other nanobodies in this class [81,94]. A similar binding mode and interaction interface can be seen for VHH U nanobody in the complex with SARS-CoV-2 S protein [88]. As a result, the binding hotspots Y369 and F377 are also preserved in the VHH U complex (Figure 6B). Structural and biophysical studies identified escape mutations in interface VHH E (G447S, Y449H/D/N, L452R, F490S, S494P/S, G496S, and Y508H) and VHH UVW (Y369H, S371P, F374I/V, T376I, F377L, and K378Q/N). Combinations of VHHs E and U, E and V, and E and W did not allow the emergence of escape mutants that were resistant to both nanobodies individually [88]. A larger interface is formed at the cryptic RBD site by VHH V nanobody, featuring stronger binding interactions and greater number of hotspots including Y369, F377, C379, Y380, V503, and Y508 (Figure 6C). The binding hotspots for VHH W nanobody corresponded to Y369, S373, and T376 residues (Figure 6D). The computed profiles are consistent with the greater binding affinity for VHH UVW nanobodies [88]. Our mutational scanning analysis also showed that escaping mutations Y369H, S371P, F374I/V, F377L, and K378Q/N at the VHH U interface resulted in considerable destabilization losses and results showed that the pattern of escaping mutations to nanobody binding may be several common dynamic sites.

In general, computational studies suggested that nanobody combinations can use binding at the cryptic binding site to establish a structurally stable regulatory “anchor” that mediates dynamic couplings between nanobody arms and provides allosteric control over structural changes in the RBM epitope [143]. Accordingly, due to avidity effects, binding of the VHH E arm at the RBM epitope may incur a smaller entropic cost and allow for local structural accommodations in these regions to compensate for the loss of binding interactions with the mutants. This suggests a plausible mechanism by which multivalent nanobodies can leverage dynamic couplings to synergistically inhibit distinct binding epitopes and suppress mutational escape. These results may prove to be useful to identify the emergent strains of concern and better understand and combat the escaping mutation mechanisms that may aid in the rational design of new vaccines and nanobody/antibody combinations.

To summarize, the analysis of computational studies of the SARS-CoV-2 S proteins and complexes with nanobodies is supplemented by mutational scanning simulations and identification of binding energy hotspots for distinct classes of nanobodies. Our results indicated that the reduced dependency induced by synergistic nanobody combinations and multivalent nanobodies on the common sites of escaping mutations may be related to the delicate balance of allosteric and avidity effects that allow for reduction of susceptibility to escape mutations. These data supported the notion that the SARS-CoV-2 S protein may exploit plasticity of specific allosteric hotspots to generate escape mutants that alter response to binding without compromising activity. This computational analysis also provided evidence that binding affinity and avidity of the SARS-CoV-2 complexes with nanobodies can be determined by structurally stable regulatory centers and conformationally adaptable hotspots that are allosterically coupled and collectively control resilience to mutational escape.

## 6. Integration of Structural and Computational Approaches for Nanobody Engineering and Avidity-Based Control of Binding Mechanisms: Outlook and Future Directions

By combining in-silico design approach and protein engineering wherein VHHs are fused to Fc domains, multi-specific nanobodies with elevated avidity and affinity as well as enhanced S/ACE2 blocking can be created. These multivalent nanobodies can be developed by knowledge of precise architectures of SARS-CoV-2 epitopes, binding modalities of the S protein of the virus, and computational screening of single nanobodies for precise control of individual epitopes. Subsequent application of computational self-assemble design tools proposed by Baker and colleagues [110] can be highly beneficial for taking in silico tools towards a direct multivalent engineering platform. Multivalent nanobody engineered systems can be either homo-valent with identical binding sites or hetero-valent with different binding sites featuring positive and negative cooperativity between system components [109]. When nanobody binding at one site leads to allosteric structural changes in the other binding site that induce the changed rate constants of the second binding event, the resulting cooperativity can be either positive, leading to the higher affinity or negative leading to the lower affinity. Recent phenomenological studies suggest that avidity and positive allosteric cooperativity can act synchronously to increase the residence time of bivalent receptor ligands [146,147]. Using a statistical mechanics model of synthetic divalent antibodies binding to HIV-1, the recent study examined the energetic effects of the linkers into the neutralization potency [148]. The strongest neutralization effects were predicted for rigid linkers that optimally span the distance between two binding sites on a trimer leading to the improved binding avidity [148]. Multivalent anti-HIV-1 therapeutics utilized avidity effects to remain potent against HIV-1 and various mutants, showing that a similar strategy of designing and probing different linkers may be viable for nanobody engineering against SARS-CoV-2 S proteins. The binding affinity and the avidity could differ in multivalent systems with linked binding sites. In some cases, low-to-medium affinity at the individual binding sites can result in a high avidity for the multivalent system due to the optimal linkage and the increased residence time in the binding sites which can be explained by the “forced proximity” of the nanobodies to their respective binding sites [146,147]. The high avidity and long residence time typical for multivalent interactions are important for the biological function of nanobodies cocktails targeting SARS-CoV-2 S proteins. As a result, it is becoming more common to combine different antibody or nanobody fragments to generate multivalent constructs that can exhibit markedly the increased target residence time when molecules can bind simultaneously to their target sites.

The biophysical characteristics of nanobodies and multivalent combinations allow for easy selection by phage display and the ability to recognize and target the unique cryptic epitopes with high specificity. These properties can be further engineered and improved using computational protein design of novel scaffolds and diverse multivalent constructs [110]. Avidity-inspired nanobody design can leverage the emerging evidence that binding affinity, avidity, and cooperativity are balanced in a complex thermodynamic mechanism of synchronous binding. The recent biophysical studies indicated that avidity-driven mechanisms may underlie functional effects of nanobody combinations and multivalent nanobody constructs to prevent viral escape, making it possible to rationally engineer a desirable level of binding specificity and design ultra-potent molecules for targeting SARS-CoV-2 S proteins. However, the thermodynamic fundamentals underlying a delicate balance of achieving high binding affinity at the individual binding sites, cooperativity and the macroscopic avidity effects are yet to be fully understood and translated into robust simulation models that would reliably predict avidity-driven binding mechanisms of nanobodies. A combination of high affinity, long residence times, and slow dissociation are the intrinsic characteristics required for the therapeutic efficacy of nanobodies because of its potentially higher target selectivity and long duration of action. The progress in nanobody engineering and discovery of highly effective therapies would require a considerable theoretical effort to translate the growing experimental data on binding affinity and avidity into robust and testable computational models that predict microscopic and macroscopic effects of binding for multivalent systems and protein assemblies. The rational nanobody engineering and future nanoparticle chemistry applications to design of novel nanobody-based therapeutics would be largely determined by the quantitative understanding of binding avidity and the effects of local environment. Although nanobodies are often superior over classical antibodies for therapeutic applications, these molecules have their own limitations and drawbacks due to rapid clearance from the body. Nonetheless, advances in protein engineering and nanoparticle chemistry have a great potential to produce next-generation nanobody constructs of higher diversity, efficacy, and fewer pharmacological side effects, ultimately leading to rational approaches transforming nanobody assemblies into high-avidity therapeutics.

## 7. Conclusions

Nanobody technology is a versatile therapeutic approach that employs powerful biophysical methods to reformat single monomeric nanobodies into multivalent format and multimerize these potent inhibitors into high-avidity therapeutics. Recent research efforts in the design, engineering, and structure-functional characterization of nanobodies and their binding with SARS-CoV-2 S proteins reflected a growing realization that nanobody combinations can exploit distinct binding epitopes and leverage the intrinsic plasticity of the conformational landscape for the SARS-CoV-2 S protein to produce efficient neutralizing and mutation resistant characteristics. Structural and computational studies have also been instrumental in quantifying the structure, dynamics, and energetics of the SARS-CoV-2 spike protein binding with nanobodies. In this review, we presented a comprehensive analysis of the current structural, biophysical, and computational biology investigations of SARS-CoV-2 S proteins and their complexes with distinct classes of nanobodies targeting different binding sites. The review provided an atomistic-level analysis of mechanisms underlying synergistic binding of nanobody combinations and multivalent nanobody constructs in combating escape mutations by leveraging binding avidity and allosteric cooperativity. Structural biology studies suggested that nanobody mixtures and rationally engineered bi-paratopic nanobody constructs offer a promising alternative to conventional monoclonal antibodies and are advantageous for controlling a broad range of infectious variants while also suppressing the emergence of virus escape mutations.

## Abbreviations

SARS	Severe Acute Respiratory Syndrome
RBD	Receptor Binding Domain
ACE2	Angiotensin-Converting Enzyme 2 (ACE2)
NTD	*N*-terminal domain
RBD	receptor-binding domain
CTD1	C-terminal domain 1
CTD2	C-terminal domain 2
FP	fusion peptide
FPPR	fusion peptide proximal region
HR1	heptad repeat 1
CH	central helix region
CD	connector domain
HR2	heptad repeat 2
TM	transmembrane anchor
CT	cytoplasmic tail
